# Critical Care Nurses Inadequately Assess SAPS II Scores of Very Ill Patients in Real Life

**DOI:** 10.1155/2012/919106

**Published:** 2012-04-02

**Authors:** Andreas Perren, Marco Previsdomini, Ilaria Perren, Paolo Merlani

**Affiliations:** ^1^Intensive Care Unit, Department of Intensive Care, Regional Hospital, 6500 Bellinzona, Switzerland; ^2^Intensive Care Unit, Department of of Anaesthesiology, Pharmacology and Intensive Care, University Hospitals and University of Geneva, 1211 Geneva, Switzerland

## Abstract

*Background.* Reliable ICU severity scores have been achieved by various healthcare workers but nothing is known regarding the accuracy in real life of severity scores registered by untrained nurses. *Methods.* In this retrospective multicentre audit, three reviewers independently reassessed 120 SAPS II scores. Correlation and agreement of the sum-scores/variables among reviewers and between nurses and the reviewers' gold standard were assessed globally and for tertiles. Bland and Altman (gold standard—nurses) of sum scores and regression of the difference were determined. A logistic regression model identifying risk factors for erroneous assessments was calculated. *Results.* Correlation for sum scores among reviewers was almost perfect (mean ICC = 0.985). The mean (±SD) nurse-registered SAPS II sum score was 40.3 ± 20.2 versus 44.2 ± 24.9 of the gold standard (*P* < 0.002 for difference) with a lower ICC (0.81). Bland and Altman assay was +3.8 ± 27.0 with a significant regression between the difference and the gold standard, indicating overall an overestimation (underestimation) of lower (higher; >32 points) scores. The lowest agreement was found in high SAPS II tertiles for haemodynamics (*k* = 0.45–0.51). *Conclusions.* In real life, nurse-registered SAPS II scores of very ill patients are inaccurate. Accuracy of scores was not associated with nurses' characteristics.

## 1. Introduction

The simplified acute physiology score II (SAPS II) [1] is probably still the most commonly used score in Europe to compare a critically ill patient's severity and—by its expanded form [2]—to evaluate clinical course and outcome [3, 4]. In addition, SAPS II has become a key-component for defining the degree of hospital reimbursement in Germany [5], and an analogous procedure is scheduled in Switzerland for the beginning of 2012 [6]. Considering the various implications, accuracy in the assessment of SAPS II scores is of the upmost importance.

 Adequate interrater reliability of SAPS II has been reported in few studies [7, 8] and small differences in values of some SAPS II variables between observers have determined important differences in scores [8]. The Acute Physiology and Chronic Health Evaluation II scoring system (APACHE II) [9] has been more extensively studied, and reliable overall APACHE II scores have been achieved by various healthcare workers (trained hospital abstractors, nurses, resident physicians, and intensivists) [10–16]. Reliability was demonstrated to further increase by training [15] as well as by a multifaceted, multidisciplinary quality improvement intervention [16]. However, these results all refer to well defined study settings with specifically trained observers, and just one study [8] has so far measured the accuracy of physician registered severity scores in real life. 

 In our intensive care units (ICU) the SAPS II score is manually assessed by specialized critical care nurses. This procedure is required exactly 24 hrs after admission or our electronic medical record system inhibits any further use for the patient in question. Assessment by nurses was chosen in order to comply with medical and organisational deficiencies (small ICUs with inexperienced junior doctors on short-term rotation and contemporaneous extra tasks about all during night shifts, no permanent ICU specialist) and because specialized nurses are present in ICUs at all hours and days and are accustomed to personally handle most of the SAPS II variables (retrieval of physiologic data and laboratory tests with their recording in the patients' charts).

 The aim of our study was (1) to assess the reliability of nurse registered SAPS II scores in real life, (2) to recognize error-prone variables, and (3) to conceive an appropriate improvement intervention.

## 2. Methods

### 2.1. Patients and Setting

This is a retrospective multicentre study, conducted within the Department of Intensive Care Medicine of the Ente Ospedaliero Cantonale, Ticino, Switzerland. Our department groups the mixed ICUs from 4 regional teaching hospitals (Bellinzona, Locarno, Lugano, and Mendrisio), has a total of 34 beds and cares for about 3,200 adult patients per year. Among the 159 nurses (with varying degrees of occupation), 70% are critical-care registered, whereas the remaining are registered nurses with ongoing specific training. Nurse/patient ratio is usually 1 : 1.5. No structured training program regarding SAPS II is offered to the nurses.

 Scoring SAPS II is performed in a semiautomatic manner: (1) manual acquisition of data: for the diagnostic information (type of admission, underlying disease variables) the nurses have complete access to the medical charts. Physiologic data (heart rate, systolic arterial pressure, urinary rate, body temperature, oxygenation status, and Glasgow Coma Scale) and laboratory findings (complete access to all variables on the electronic medical record system) are consecutively documented by nurses on the daily patient survey charts, from which they are ultimately retrieved for registration of the SAPS II score. (2) For every variable the nurse has to select the most pondered option (among the lowest and highest value), that is eventually entered in the electronic medical record system. Consecutively, this system automatically calculates the final score. Identification of the nurse-recorder is assured by means of a personal code.

 Patients ≥ 18 years of age, admitted to our ICUs between January 2010 and October 2010, were eligible. Considering the retrospective, noninterventional design of this quality assurance study, no informed consent was required by the Cantonal Ethics Committee.

### 2.2. Study Protocol

Among 2386 eligible patients the primary investigator randomly selected 30 patients per ICU presenting with the following principal discharge diagnostics (number of patients): septic shock (5), acute ischemic stroke (3), acute myocardial infarction (3), cardiopulmonary arrest (3), acute heart failure (3), acute respiratory failure due to pneumonia (3), chronic obstructive pulmonary disease (2), acute pancreatitis (2), polytrauma (2), arrhythmias (2), and patients with an ICU stay less than 24 hrs (2). Patients' charts were then obtained by employees of the corresponding local quality control services and collocated for the review “in loco.”

 Two experienced, board-registered intensivists and one critical-care registered nurse specifically trained for the use of SAPS II created a structured form for review that was principally based on the original definitions of the variables necessary for SAPS II [1]. The following issues were more accurately specified in order to correctly reflect organ dysfunction: (1) in case of uninterrupted vasopressor therapy for haemodynamic instability during the first day, the definitions were adapted according to elements proposed in the SOFA score [17], (2) cardiac arrest leading to ICU admission was deemed equal to cardiac arrest within ICU in order to ponder the increased mortality; (3) utilisation of laboratory tests performed immediately prior to ICU admission was permitted, as follow-up tests within our ICUs are generally executed by a careful and selective approach; (4) sensory and motor aphasia due to acute ischemic stroke in a patient with otherwise adequate mentation were disregarded for the calculation of the Glasgow Coma Scale.

### 2.3. Data Collection and Evaluation

The analysis was done by the three investigators by means of the above-mentioned template. The review process was performed in two steps. During the first stage the investigators independently examined the charts from all 30 patients and assessed the SAPS II scores. The results were evaluated, differences between the reviewers' judgments were eventually resolved by discussion, and a final consensus (gold standard) was achieved. The second step served for assessment of agreement between the nurse-registered SAPS II scores (retrieved from the electronic medical record system by the primary investigator) and the gold standard.

 This procedure was repeated in all four ICUs for a total of 120 patients. For each patient the following data were registered: (1) SAPS II sum score, (2) every item of the SAPS II score, (3) differences in the reviewers' judgements and (4) differences between the nurse registered SAPS II score and the gold standard. The following variables were retrieved for the nurses that did the SAPS II scoring: centre, gender, certification, and duration of specific professional experience.

### 2.4. Statistical Analysis

Variables are expressed as mean ± standard deviation (SD) if not specified otherwise. A *P* < 0.05 was considered statistically significant. All analyses were performed with Stata statistical software, release 11.0 (Stata Corporation, College Station, TX, USA) and Statview (SAS institute Inc., Cary, NC, USA).

#### 2.4.1. Validation of the Gold Standard

Agreement between reviewers was assessed by average measure interclass correlation coefficient (ICC) (Spearman-Brown correction) for continuous variables (sum scores) and with weighted kappa statistics (and 95% confidence interval) for analysis of the different SAPS II items. Kappas were calculated only for items where more than 20% of the values differed from baseline [18]. Mean agreement for the sum scores and for items between reviewers was assessed by calculating their mean percentage of identical classifications among a pair of reviewers. Perfect agreement was defined as identical categorization of sum scores and items. Differences between the reviewers were analyzed according to the SAPS II tertile (low, medium, and high) and according to their mechanism.

#### 2.4.2. Comparison of the Nurse-Assessed SAPS II Scores and the Gold Standard

Differences in the sum-scores were assessed by a paired *t*-test. The mean difference (with 95% CI) and the mean absolute difference (i.e., the mean of the value of the difference) between SAPS II sum scores (gold standard minus nurses) were calculated.

 Agreement between nurses and the gold standard was assessed as between the reviewers. Agreement was defined as identical categorization of sum scores and items. Kappas and the agreement were analyzed according to the SAPS II tertile (low, medium, and high) and the ICC of the sum scores were analyzed according to the SAPS II tertile and to the center. Concerning the SAPS II sum score a modified Bland and Altman analysis with on the *x*-axis the gold standard and on the *y*-axis the difference between the two sum scores (gold standard minus nurse value) was performed, completed by a regression analysis between the SAPS II gold standard and the SAPS II gold standard minus the nurse value sum score. A scatter plot between the difference in the predicted mortality calculated with the SAPS II gold-standard sum-score minus the mortality predicted by the SAPS II nurse-registered sum score (on the *y*-axis) and the SAPS II gold standard sum score (on the *x*-axis) was performed. The difference between the predicted mortalities (deriving from SAPS II sum-scores: gold-standards minus nurse-assessed values) was modelized, using the formula identified in the regression analysis described above.

 A univariate analysis was done to define risk factors for the occurrence of an error in items or sum scores, including centers and nurse characteristics (gender, professional experience, and certification). Results are shown as odds ratios (OR; 95% CI) in order to estimate the effect size of risk factors associated with an erroneous estimation. A multivariate logistic regression was performed in order to obtain adjusted estimates of the ORs and to identify factors independently associated with errors, including for the model always the 3 nurse variables and the 4 centers. The multivariate analysis was performed only for those items with sufficient errors enabling the analysis: assuming that for each of the 6 considered predictor variables (centres and nurse characteristics) about 5–10 events should be available, we needed a minimum of 30 and a maximum of 90 errors.

## 3. Results

### 3.1. Gold Standard Created by Reviewers

A total of 120 different SAPS II scores (1800 variables) were assessed and for 171 cases of divergence (9% of all variables) a gold standard had to be defined by consensus. The minimum-maximum (median) gold standard SAPS II score overall, of low, medium, and high SAPS II tertiles was 6–111 (38), 6–31 (22), 32–47 (38), and 48–111 (70), respectively. Agreement for sum scores among reviewers was almost perfect (mean ICC = 0.985; significant correlation *P* < 0.0001; *P* for significant difference > 0.05).  Table 1 shows the reviewers' reliability regarding the single variables assessed; accuracy was highest for temperature and bilirubin (perfect agreement = 1.0 and 0.99, resp.) and lowest for systolic blood pressure (perfect agreement = 0.75). Errors in reviewers' assessment (Table 2) were most frequently observed in the high SAPS II tertile (79 errors), followed by the medium (52) and low tertiles (40). Occurrence of errors was basically due to negligence (49% of cases), followed by a problem related to the definition of the variable (22%), incorrect calculation (16%), and others (13%). Table 2 lists the differences between the reviewers' judgments according to the kind of error.

### 3.2. Accuracy of Nurse-Registered SAPS II Scores

The mean (±SD) nurse registered SAPS II sum-score was 40.34 ± 20.19 points versus 44.17 ± 24.86 points of the gold standard (*P* = 0.002). About 90% of the SAPS II sum-scores (112/120) were erroneous in at least one variable (87.5% (35/40) in the low, 97.5% (39/40) in the medium, and 95% (38/40) in the high SAPS II tertiles).  Table 3 shows the accuracy in assessment of the single variables when compared to the gold standard. Overall, there was good agreement in the variables sodium, temperature, age, chronic diseases, leucocytes, potassium, and bilirubin (0.83–0.97); the lowest agreement was found in heart rate and systolic pressure (0.45–0.51). Calculated kappas were best for age and lowest for heart rate and systolic pressure (0.32–0.37). Generally, agreement and Kappas were worst in the high SAPS II tertile.

**Figure 1:**
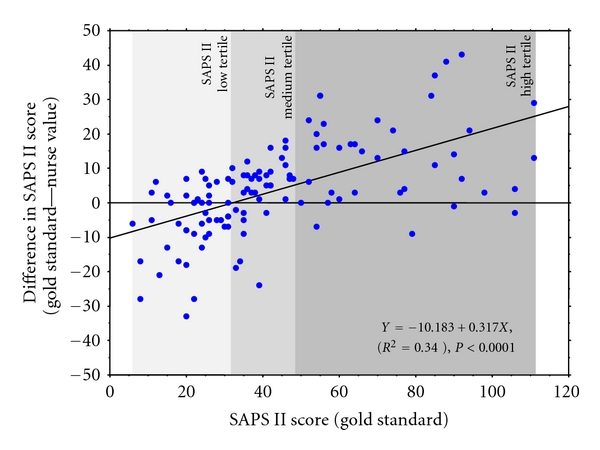
Linear regression between the difference (gold standard-nurse value) of the SAPS II sum-score and the gold-standard SAPS II sum-score. SAPS II tertiles are illustrated.

 Although SAPS II sum scores were underscored throughout the whole range, there were considerable differences among SAPS II tertiles, in bias and bias dispersion of the difference (SD of difference) and minimum and maximum differences (Table 4). Differences (absolute differences) changed also depending on the SAPS II tertile.  Table 5 shows the origin of the over- and underestimation of the low and high SAPS II sum score tertiles.  Figure 1 confirms a general trend to overestimate low (≤ 32 points) and underestimate higher sum scores, by highlighting a significant regression between the difference and the gold standard SAPS II sum score (regression of the Bland and Altman analysis: *y* = −10.183 + 0.317*x; *R* ^2^ = 0.34, *P* < 0.0001). The cut-off point between over- and underestimation was at 32 SAPS II gold standard points.

**Figure 2:**
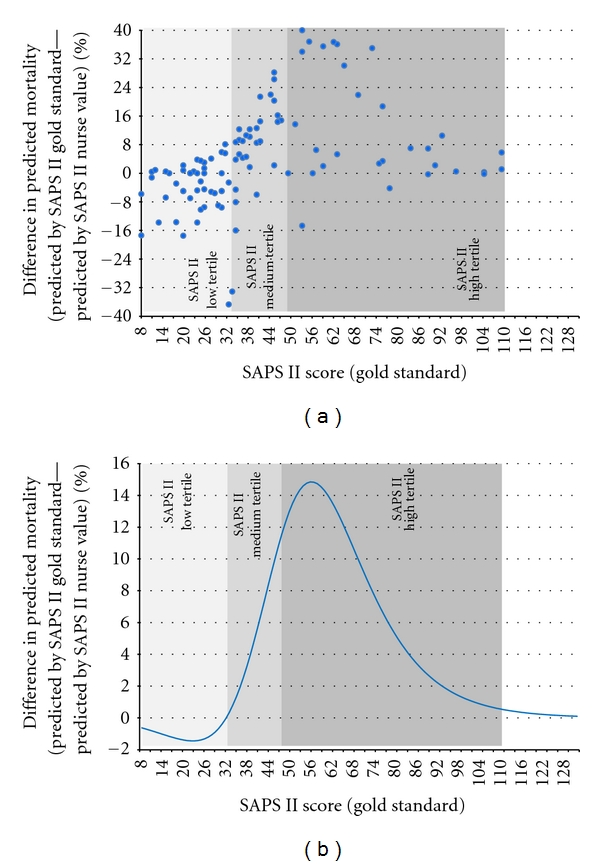
(a) : Scatter plot with the difference in the predicted mortality, (b) : Modelization of the difference in the predicted mortalityIn both figures the SAPS II tertiles are illustrated.

 The mean nurse-predicted mortality rate was 29.11 ± 28.65% versus 35.39 ± 33.59% of the gold standard (*P* = 0.002). The mean difference between the predicted mortality by the gold standard and the predicted mortality by nurses was 6.28% (CI −32.9 to 45.5%, range −50.7 to 56.9%) and a mean absolute difference of 13.8% (CI 0.0 to 30.6%, range 0 to 56.9%). Figures 2(a) (scatter plot) and 2(b) (provisional modelization) illustrates the over- and underestimation of the predicted mortality depending on the SAPS II (golden standard) sum-score values. Considerable differences were found in bias and bias dispersion of the difference (SD of difference) and minimum and maximum differences among the different centers (Table 4).

Table 5 illustrates the variables that induce the overestimation of lower SAPS scores (oxygenation, urinary output, urea, bicarbonate, and bilirubin) and underestimation of the highest SAPS II scores (heart rate, systolic blood pressure, urea, and Glasgow Coma Scale). 

 A total of 78 nurses registered the 120 SAPS II scores. No association was found by univariate and multivariate analysis between nurses' characteristics (experience, certification, gender, and centres) and erroneous scoring of the total SAPS II score or its variables.

## 4. Discussion

Our study shows that nurse-registered SAPS II sum scores are quite inaccurate. Overall, there was a clear overestimation of lower SAPS II scores and an underestimation of higher SAPS II scores with a center-tendency trend (one fits all tendency). Larger absolute errors were performed in the higher scores. Overall haemodynamics were the most error-prone variables and mistaken assessment was independent of the nurses' characteristics. However, in the higher SAPS II tertiles, haemodynamics as well as urea and the Glasgow Coma Scale contributed to the underestimation whereas in the lower SAPS II tertile errors in the oxygenation status, urinary output, urea, bicarbonates, and the bilirubin concentration contributed to overestimation of the SAPS II sum scores.

 Astonishingly, the agreement of haemodynamic variables—although apparently simple—was inadequate. Our results are comparable to those from Strand et al. [7], who reported similar difficulties for Norway junior doctors in assessing heart rate and systolic blood pressure. A mathematical explanation of this problem could be that five (four) choices are given for scoring of systolic blood pressure (heart rate) whereas the rating of the other physiological variables is generally less demanding. Another explication may be that there it is not only to chose the quantity of deviation (from the normal value) but also the direction of highest ponderation (lowest versus highest value). 

 With this retrospective audit we were not able to disclose by which mechanisms nurses created mistakes in assessing the SAPS II scores. However, we could show that professional experience and certification had no impact on the occurrence of errors, neither was there a general centre effect. The analysis of the three reviewers' most frequent sources of problems in defining the gold standard might give some insight (Table 2). In this sense, negligence was the most common source of erroneous assessment. Problems related to the definition of the variables and incorrect calculation of data (oxygenation ratio, urinary output, age) as well as lacking interest in scoring should also be considered. It is important to emphasize that our nurse-registered SAPS II scores are based on manual acquisition of data. The nurses rely on previously registered physiological data from the daily patient survey charts and administrative data from the physician charts. They eventually insert manually the variables in the electronic medical record system that automatically calculates the final score. SAPS II being a severity score concerning the first 24 hrs after ICU admission, several care givers are involved in the collection of the different variables and each of them is prone to errors. 

 Both, reviewers and nurses, globally underestimated SAPS II scores. Most interestingly, we found a negative relationship between the height of the nurse registered sum-scores and their reliability, when compared to the gold standard: the higher the sum scores the more they were underestimated. Exclusion of critical pre-ICU data (e.g., cardiac arrest) may seriously affect SAPS II scores and predicted mortality, as much as some pathologic data goes unconsidered (11 points for heart rate; 13 and 26 points for systolic blood pressure and Glasgow Coma Scale, resp.). The same might apply, although to a smaller extent, for mistaken omission of pathologic laboratory findings, obtained immediately prior to ICU admission (e.g., in the emergency room, on the ward).

 The analysis of the correlation and agreements between the nurse-assessed SAPS II scores and the gold standards, calculated without considering the pre-ICU data, showed only slightly better results (not shown). The impact of the differences in scoring (over- and underestimations) may be important. Indeed, we can identify at least 3 areas of concern. First, the stratification or adjustments for research purposes on the basis of routinely (nurse-) assessed SAPS II scores (particularly in multicenter studies with the support of different systems) could be misleading. Secondly, benchmarking across ICUs may be heavily biased. Finally, reimbursements based primarily or secondarily on the SAPS II score as in Germany or Switzerland [5, 6] may seriously suffer from the inaccuracy of the SAPS assessment, especially by the underestimation of higher SAPS II scores. Indeed, in an European study 10% (12%) of respondents reported that their reimbursement relied primarily (secondarily) on severity scores [19].

 It has been shown that automatic retrieval of variables may increase scores through a higher sampling rate [20]. Such an approach would probably also decrease the number of missing components who otherwise may lead to an underestimation of sum scores and predicted mortality [21]. A correct transmission of pertinent data, if properly validated, could also increase reliability. In this sense we are adapting our electronic medical record system in order to automatically prepopulate the SAPS II scores with laboratory results and age. Furthermore, by means of a data management system, achievement of haemodynamic and respiratory variables could be automatized. This system, however, is also prone to different problems. First, importation of incoherent data may occur if the information is not manually verified. Second, as severity scores were developed and calibrated with manually acquired data, computer-assisted extraction of data may alter outcome prediction [22]. Accurate acquisition and correct transmission of related data are definitely essential, but without adequate knowledge of the definitions and their exact application, SAPS II scores will hardly become very reliable. Thus, a structured training program will be implemented in our department in order to increase understanding and motivation. Furthermore, the introduction of an interactive program asking in detail the highest and lowest value of a variable (maybe also requiring the exact data) may optimize the SAPS II assessment reducing some of the errors called “negligence.”

 Our study presents some strengths and/or limitations: (1) scoring is a difficult task, even for specifically trained reviewers. By consequence, one might question our gold standard. Actually, we believe that this point represents a strength. The way we did this audit (see Section 2) actually excluded any bias regarding professional background, specific training for SAPS II, and assessment practice. Ultimately, there was excellent agreement among reviewers regarding the sum scores. Analysis of the different subscores revealed almost perfect agreement for most of the variables and still substantial agreement for systolic blood pressure, urinary output and the Glasgow Coma Scale. Moreover, the multicentre design of this study permits a certain generalization of the results. (2) The introduction of adapted definitions regarding haemodynamic instability (see Section 2) might have influenced our results. However, exact analysis of the variable systolic blood pressure revealed that only in about 30% of cases there was an underscoring due to disregarding of continuous vasopressor therapy. Moreover, we believe that the definition of this variable should be changed. In order to detect an increased risk of mortality it seems not adequate to score patients with normal systolic blood pressures under huge amounts of vasopressors as “regular.” (3) One might also criticize our real-life situation, where nurses do the assessment of SAPS II scores. However, there are no unequivocal data in the literature able to confute our method. In the unique study directly comparing residents with nurses there was no significant difference between mean APACHE II scores or mean predicted mortality rates [10]. On the other hand, accuracy of scoring among physicians was reported to depend rather on instruction [15] than on the professional experience [23]. (4) Finally, generalization of our results might be further limited inasmuch they refer to SAPS II, whereas the most frequently used ICU severity score worldwide is the Acute Physiology and Chronic Health Evaluation (APACHE) II score [9]. However, we would like to emphasize that the two severity scores diverge principally in the attribution of points for different degrees of organ dysfunction and much less in the choice of the requested items (e.g., age and most physiological variables are superimposable).

 In conclusion, our study suggests that untrained critical care nurses inadequately assess SAPS II scores in real life and that reliability was not influenced by different backgrounds, levels of training and gender. Higher SAPS II sum scores are underestimated and lower scores overestimated. These differences may severely impact on benchmarking, research results, and ICU reimbursement. A multifaceted improvement intervention [16], based on automatic (computer-based) retrieval of most physiological data and implementation of a structured training program, is warranted. Whether these observations may apply also to other severity scores or other healthcare professionals remains an interesting question to be answered.

##  Disclosure 

This work was performed at the four regional teaching hospitals of Southern Switzerland: Bellinzona, Locarno, Lugano and Mendrisio.

## Figures and Tables

**Table 1:** Reliability across reviewers for the single variables of the SAPS II score.

Variable	Kappa^a^ (95% CI)	Mean agreement^b^	Perfect agreement^c^
Heart rate	0.84 (0.79–0.89)	0.88	0.83
Systolic blood pressure	0.76 (0.68–0.84)	0.83	0.75
Temperature	NA	—	1.0
Oxygenation	0.84 (0.80–0.88)	0.91	0.87
Urinary output	0.76 (0.72–0.80)	0.90	0.86
Urea	0.94 (0.91–0.97)	0.97	0.95
Leucocytes	NA	—	0.96
Potassium	NA	—	0.88
Sodium	NA	—	0.98
Bicarbonate	0.84 (0.81–0.87)	0.93	0.89
Bilirubin	NA		0.99
Glasgow Coma Scale	0.74 (0.69–0.79)	0.88	0.82
Age	0.94 (0.92–0.96)	0.95	0.93
Chronic diseases	NA	—	0.94
Type of admission	NA	—	0.94

^a^Mean weighted Kappa (95% confidence interval) of the 3 reviewer.

^b^Mean proportions of agreement among the 3 reviewers versus gold standard.

^c^Percentage of total agreement among the 3 reviewers versus gold standard.

NA: not applicable; no reliable Kappa statistics (≤20% of results differ from norm).

**Table 2:** Differences among the reviewers' judgments for the different items according to the SAPS II tertile and the mechanism of error.

Variables	Cases				Mechanism of error			
	Overall	Low SAPS (6–31 points)	Medium SAPS (32–47 points)	High SAPS (48–111 points)	Overall			
	*N* = 120	*N* = 40	*N* = 40	*N* = 40	Differences=171/Scores=120			
*N*, % of differences, % of scores	*N*, (%/%)	*N* (%,%)	*N* (%,%)	*N* (%,%)	Definition^a^ (*n*)	Calculation^b^ (*n*)	Negligence^c^ (*n*)	Others^d^ (*n*)
Heart rate	21 (12/17.5)	8 (20/20)	6 (12/15)	7 (9/17.5)	5	0	14	2
Systolic blood pressure	30 (18/20)	9 (22.5/22.5)	13 (25/32.5)	8 (10/20)	9	0	19	2
Temperature	0	0	0	0	0	0	0	0
Oxygenation	16 (9/13)	2 (5/5)	4 (8/10)	10 (13/25)	1	8	3	4
Urinary output	17 (10/14)	4 (10/10)	7 (13.5/17.5)	6 (7.5/15)	1	12	2	2
Urea	6 (4/5)	1 (2.5/2.5)	3 (6/7.5)	2 (2.5/5)	0	0	5	1
Leucocytes	5 (3/4)	0	1 (2/2.5)	4 (5/10)	0	0	5	0
Potassium	14 (8/12)	2 (5/5)	4 (10)	8 (10/20)	0	0	11	3
Sodium	3 (2/2.5)	0	0	3 (4/7.5)	0	0	3	0
Bicarbonate	13 (8/11)	2 (5/5)	4(8/10)	7 (9/17.5)	1	0	8	4
Bilirubin	1 (0.5/1)	1 (2.5/2.5)	0	0	0	0	1	0
Glasgow Coma Scale	22 (13/18)	7 (17.5/17.5)	3 (6/7.5)	12 (15/30)	8	0	11	3
Age	9 (5/7.5)	2 (5/5)	3 (6/7.5)	4 (5/10)	0	8	1	0
Chronic diseases	7 (4/6)	1 (2.5/2.5)	2 (4/5)	4 (5/10)	5	0	2	0
Type of admission	7 (4/6)	1 (2.5/2.5)	2 (4/5)	4 (5/10)	7	0	0	0

*N* (%)								
Total of differences	171 (100)	40 (100)	52 (100)	79 (100)	37 (22)	28 (16)	85 (49)	21 (13)
Total of scores with differences	94 (78)	29 (72.5)	30 (75)	35 (87.5)	—	—	—	—

^a^Problem related to the definition of variables and its application (e.g., chronic diseases, type of admission, sustained haemodynamics, GCS in aphasic stroke).

^b^Mathematical problem (e.g., oxygenation ratio, age, daily urinary output).

^c^Insufficient examination of the charts (e.g., erroneous exclusion of laboratory results).

^d^Other mechanism (e.g., insufficient available data in the chart).

**Table 3:** Reliability of nurses versus gold standard SAPS II items in overall SAPS II and SAPS II tertiles.

Variable	Kappa^a^ (95% CI)				Agreement^b^			
	Overall	Low SAPS II (6–31 points)	Medium SAPS II (32–47 points)	High SAPS II (48–111 points)	Overall	Low SAPS II (6–31 points)	Medium SAPS II (32–47 points)	High SAPS II (48–111 points)
Heart rate	0.32 (0.16–0.48)	0.28 (0.12–0.44)	0.32 (0.15–0.49)	0.22 (0.08–0.36)	0.45	0.55	0.47	0.35
Systolic blood	0.37 (0.24–0.50)	NA	0.22 (0.06–0.38)	0.20 (0.08–0.32)	0.51	0.63	0.50	0.40
pressure								
Temperature	NA	NA	NA	NA	0.95	1.00	0.97	0.87
Oxygenation	0.66 (0.56–0.76)	0.33 (0.16–0.50)	0.72 (0.60–0.84)	0.54 (0.40–0.68)	0.71	0.87	0.70	0.55
Urinary output	0.77 (0.70–0.84)	0.73 (0.63–0.83)	0.80 (0.70–0.90)	0.73 (0.64–0.82)	0.58	0.52	0.67	0.55
Urea	0.71 (0.64–0.78)	0.70 (0.62–0.78)	0.71 (0.62–0.80)	0.71 (0.63–0.79)	0.67	0.57	0.75	0.67
Leucocytes	NA	NA	NA	0.40 (0.29–0.51)	0.83	0.92	0.85	0.72
Potassium	NA	NA	0.39 (0.24–0.54)	0.32 (0.19–0.45)	0.83	0.95	0.82	0.72
Sodium	NA	NA	NA	NA	0.97	0.97	0.97	0.95
Bicarbonate	0.81 (0.76–0.86)	0.77 (0.70–0.84)	0.77 (0.69–0.85)	0.76 (0.65–0.87)	0.68	0.62	0.85	0.55
Bilirubin	NA	NA	NA	NA	0.83	0.75	0.87	0.85
Glasgow Coma Scale	0.80 (0.71–0.89)	0.48 (0.19–0.77)	0.68 (0.43–0.92)	0.89 (0.80–0.98)	0.78	0.85	0.77	0.72
Age	0.98 (0.97–0.99)	0.98 (0.96–1.00)	0.99 (0.97–1.00)	0.97 (0.94–1.00)	0.93	0.92	0.95	0.92
Chronic diseases	NA	NA	NA	NA	0.93	0.97	0.92	0.87
Type of admission	NA	NA	NA	NA	0.78	0.85	0.77	0.70

^a^Mean weighted Kappa of the 120 nurse-registered SAPS II scores versus the gold standard.

^b^Mean proportions of agreement of the nurses versus the gold standard.

NA: not applicable; no reliable Kappa statistics (≤20% of results differ from norm).

**Table 4:** Agreement of nurse assessed SAPS II sum scores according to SAPS II tertiles and to the ICU site.

	ICC	ΔSAPS II (GS—nurses)				Absolute ΔSAPS II (GS—nurses)			
		Mean	SD	Minimum	Maximum	Mean	SD	Minimum	Maximum
Overall	0.81	3.8	13.5	−33	43	10.4	9.3	0	43
Low SAPS II ( 6–31)	0.60	−5.9	10.1	−33	9	8.4	8.0	0	33
Medium SAPS II (32–47)	0.54	3.3	9.1	−24	18	8.0	5.4	0	24
High SAPS II (48–111)	0.77	14.0	13.0	−9	43	15.0	11.7	0	43
Center A	0.81	1.2	12.3	−28	31	8.5	8.9	0	31
Center B	0.77	5.3	16.9	−28	43	14.4	9.9	3	43
Center C	0.76	2.6	15.5	−33	41	11.4	10.6	0	41
Center D	0.89	6.0	8.9	−13	31	8.1	7.0	0	31

ICC: interclass correlation coefficient between gold standard and nurses.

Δ SAPS II: difference in SAPS II scores between gold standard and nurses.

SD: standard deviation.

**Table 5:** Mean differences between the gold standards and nurse-assessed SAPS II scores concerning the values of the different items composing the SAPS II score, overall, and by tertiles.

Variable		Mean difference (gold standard − nurse value)		
	Overall	Low SAPS II (6–31 points)	Medium SAPS II (32–47 points)	High SAPS II (48–111 points)
Heart rate	2.0	0.6	1.5	4
Systolic blood pressure	2.8	0.6	2	5.8
Temperature	0.1	0	−0.1	0.2
Oxygenation	−0.2	−0.7	−0.3	0.45
Urinary output	−1.3	−1.5	−0.9	−1.5
Urea	−0.1	−2.6	1.2	1.2
Leucocytes	0.1	0.1	−0.2	0.4
Potassium	0.4	0.0	0.5	0.5
Sodium	0.0	0.0	0.0	0
Bicarbonate	0	−0.8	0.2	0.5
Bilirubin	−0.6	−1	−0.5	−0.3
Glasgow Coma Scale	1.2	0.0	0.2	3.6
Age	0.0	0.0	−0.1	0
Chronic diseases	−0.1	−0.2	0.3	−0.4
Type of admission	−0.4	−0.4	−0.4	−0.4

ΔSAPS II sum scores	3.8	−5.9	3.3	14.0
